# From mitochondrial zonation to immune dysregulation: a mechanistic axis in diabetic cardiomyopathy

**DOI:** 10.3389/fcvm.2026.1775592

**Published:** 2026-05-19

**Authors:** Jiahui Chen, Linghua Fu, Jing Zhang, Rui Ding, Hanbin Zhang, Gaosi Xu, Jinzhu Hu, Pingping Yang

**Affiliations:** 1 Department of Endocrinology and Metabolism, The Second Affiliated Hospital, Jiangxi Medical College, Nanchang University, Nanchang, China; 2 HuanKui Academy, Nanchang University, Nanchang, Jiangxi, China; 3 Department of Cardiovascular Medicine, The Second Affiliated Hospital, Jiangxi Medical College, Nanchang University, Nanchang, Jiangxi, China; 4 Department of Anesthesiology, The Second Affiliated Hospital, Jiangxi Medical College, Nanchang University, Nanchang, China; 5 The Second Clinical Medical School, Jiangxi Medical College, Nanchang University, Nanchang, Jiangxi, China; 6 Department of Nephrology, The Second Affiliated Hospital, Jiangxi Medical College, Nanchang University, Nanchang, Jiangxi, China

**Keywords:** diabetic cardiomyopathy, innate immunity, mitochondrial heterogeneity, mitochondrial reactive oxygen species, mitophagy, myocardial fibrosis

## Abstract

Diabetic cardiomyopathy (DbCM) is characterized by early diastolic dysfunction, myocardial fibrosis, and progressive energetic failure, in which mitochondria dysfunction have a central role. Although mitochondrial dysfunction is well established in DbCM, emerging spatially resolved data indicate that cardiomyocytes contain functionally distinct mitochondrial subpopulations with differential susceptibility to metabolic stress. In this Review, we synthesize mechanistic and translational evidence and propose a unifying, testable hypothesis. Selective remodeling of membrane lipids and cristae destabilization may render specific mitochondrial subsets “early-damaged.” These mitochondria produce sustained mitochondrial reactive oxygen species (mtROS), release oxidized mtDNA or mitochondrial-derived vesicles (MDVs), and subsequently activate innate immune pathways. We particularly emphasize distinct mitochondrial subpopulations, including subsarcolemmal (SSM), interfibrillar (IFM), and perinuclear mitochondria (PNM). Finally, we posit a proof-of-concept translational roadmap involving biomarker-guided, spatially informed preclinical endpoints and targeted interventions. Conceptualizing DbCM as a disease of mitochondrial heterogeneity and maladaptive mtROS–mtDNA–innate immune coupling reorients therapeutic strategy from global antioxidant suppression toward precision, organelle- and location-specific modulation.

## Introduction

Diabetic cardiomyopathy (DbCM) is defined as myocardial structural and functional abnormalities in patients with diabetes, independent of coronary artery disease, hypertension, or other common cardiac conditions (1). In contrast to primary cardiomyopathies such as hypertrophic cardiomyopathy and dilated cardiomyopathy, DbCM is a secondary cardiomyopathy, representing the localized cardiac involvement of diabetes as a systemic disease (2). Early clinical manifestations include impaired diastolic relaxation, reduced myocardial compliance, and altered energy metabolism. As the disease advances, these abnormalities progress to heart failure, often with a phenotype dominated by heart failure with preserved ejection fraction (HFpEF), which is increasingly recognized in diabetic populations (3, 4).

Mitochondria are crucial in the pathogenesis of DbCM, owing to their essential role in sustaining the high energy demand of heart. Beyond ATP production, mitochondria actively shape cellular responses through the generation of mitochondrial reactive oxygen species (mtROS) and regulation of calcium handling, thereby linking metabolic stress to structural remodeling and functional decline. Traditionally, mitochondrial dysfunction in DbCM has been considered as a global organelle impairment. However, accumulating evidence suggests that this view is overly simplistic (5, 6), as it fails to explain why myocardial injury often arises in spatially restricted regions, why fibrosis develops focally rather than diffusely, or why specific subsets of cardiomyocytes appear disproportionately vulnerable.

Cardiomyocytes harbor distinct mitochondrial subpopulations, including subsarcolemmal (SSM), interfibrillar (IFM), and perinuclear mitochondria (PNM), which differ in respiratory capacity, calcium sensitivity, susceptibility to mtROS, and quality-control mechanisms such as biogenesis and mitophagy. This spatial and functional heterogeneity provides a framework to explain why pathological changes in DbCM, such as focal fibrosis, microvascular dysfunction, and cell death, often display regional distribution within the myocardium (7, 8). Nevertheless, how these heterogeneous mitochondrial pools contribute to disease initiation, progression or clinical phenotypes remains poorly defined.

In parallel, the understanding of mtROS has shifted from being solely a source of oxidative damage to a more nuanced role as signaling mediators. At low to moderate levels, mtROS participate in adaptive responses such as metabolic switching, redox signaling, and selective mitophagy (9, 10). Conversely, excessive or prolonged mtROS generation drives maladaptive remodeling, fibrosis, and functional impairment. Thus, the pathological impact of mtROS is determined not only by its absolute quantity but also by its temporal dynamics and subcellular localization (11). Yet the precise link between mitochondrial heterogeneity, localized ROS signaling, and subsequent immune or fibrotic responses has not been systematically integrated.

Here, we integrate emerging concepts of mitochondrial heterogeneity with the context-dependent nature of mtROS signaling, proposing a unified framework that explains how selective mitochondrial vulnerability and spatiotemporal ROS dynamics jointly drive localized myocardial injury and clinical phenotypes in DbCM. This perspective not only highlights unresolved mechanisms but also outlines new experimental and translational avenues.

## Mitochondrial heterogeneity in diabetic cardiomyopathy

Mitochondrial heterogeneity in the myocardium, defined as the coexistence of structurally, molecularly, and functionally distinct mitochondrial subpopulations within a single cardiomyocyte (8, 12), represents not merely a descriptive phenomenon, but a mechanistically grounded framework that helps explain the spatial initiation and progressive propagation of energetic failure in DbCM.

This heterogeneity arises from microdomain-specific differences in mitochondrial biogenesis, protein import, substrate access, and organelle tethering (13), which collectively impose distinct metabolic and signaling constraints on subsarcolemmal mitochondria (SSM), interfibrillar mitochondria (IFM), and perinuclear mitochondria (PNM). For example, SSM reside beneath the sarcolemma and are preferentially exposed to fluctuations in extracellular substrates, ion fluxes, and membrane-associated signaling complexes (8, 14). In contrast, IFM are tightly packed between myofibrils, physically coupled to ATP-consuming contractile machinery, and exhibit a more stable structural integration with cytoskeletal elements and mitochondrial reticulum networks, which may enhance metabolic efficiency and resistance to acute stress (15).

At the molecular level, these spatial differences are reinforced by distinct proteomic and lipidomic signatures, including variations in cardiolipin composition, respiratory chain supercomplex assembly, and the abundance of fatty-acid oxidation enzymes and mitochondrial calcium uniporter (MCU) components (16, 17). Such differences directly influence electron transport efficiency, ROS generation, and Ca^2+^ buffering capacity, thereby creating subpopulation-specific thresholds for dysfunction.

Epidemiological evidence shows that the prevalence of diabetes in female patients with heart failure is significantly higher whether the left ventricular ejection fraction is reduced or preserved (18); in turn, multiple clinical studies have confirmed a higher cardiovascular disease risk in female vs. male patients with diabetes (19, 20). Accumulating clinical and preclinical evidence confirms sex-specific differences in mitochondrial subcellular heterogeneity (21), which may be key contributors to the higher prevalence of DbCM in women. Estrogen receptors are present in myocardial mitochondria (22, 23). Through these mitochondrial estrogen receptors, estrogen exerts innate cardiovascular protection in women by maintaining mitochondrial homeostasis (24, 25). However, in the pathological environment of diabetes characterized by high glucose, insulin resistance and lipotoxicity, the protective effects of estrogen are substantially attenuated (26, 27). In parallel, diabetes directly increases the apoptotic susceptibility of interfibrillar mitochondria (IFM) via enhanced oxidative stress (28, 29). Notably, under physiological conditions, the ADP/O ratio, a measure of oxidative phosphorylation efficiency, is significantly higher in female IFM than in male IFM (21). While this higher efficiency may confer an advantage under normal metabolic conditions, it could predispose female IFM to greater injury upon diabetic insult, as a higher membrane potential often facilitates reactive oxygen species generation and mitochondrial permeability transition pore opening (30). Therefore, the conjunction of diabetes-induced loss of estrogen-mediated protection and the distinct metabolic phenotype of female IFM offers a plausible explanation for the greater DbCM burden in women.

In the context of type 2 diabetes, converging evidence from both animal models and human myocardium indicates that SSM undergo earlier and more pronounced impairments in morphology, membrane potential, and oxidative phosphorylation, with particular deficits in complex I activity and fatty-acid-supported respiration (31, 32). This selective vulnerability is thought to arise from their anatomical proximity to the sarcolemma, where they are more directly exposed to lipotoxic intermediates and oxidative stress, coupled with a relatively limited antioxidant defense capacity and a less integrated position within the mitochondrial reticular network (8). As a result, their ability to buffer and redistribute localized damage is constrained. Functionally, this “subsarcolemmal-first” pattern of injury is especially detrimental, as it compromises ATP supply to membrane ion pumps and channels, ultimately leading to impaired sarcolemmal excitability and disrupted Ca^2+^ handling.

Conversely, in type 1 diabetes models, IFM display more pronounced alterations at the proteomic level, including downregulation of fatty-acid oxidation enzymes, electron transport chain subunits, and components of the mitochondrial protein import machinery (e.g., TOM/TIM complexes) (29, 33). This suggests that insulin deficiency and altered substrate utilization may preferentially perturb mitochondrial populations that are more dependent on tightly regulated oxidative metabolism and protein turnover. Importantly, impaired protein import and turnover could lead to the accumulation of misfolded proteins and defective respiratory complexes, thereby amplifying mitochondrial dysfunction within IFM (34), despite their structurally protected localization. The mitochondrial responses to diabetes described above are not universally consistent across all models. Rather, they exhibit model-dependent patterns: in type 2 diabetes, SSM display earlier and more pronounced functional impairments due to lipotoxic and oxidative stress exposure (31, 32), whereas in type 1 diabetes, IFM show more prominent proteomic alterations linked to insulin deficiency and impaired protein turnover (33). Thus, the nature of the diabetic model shapes the preferential vulnerability of distinct mitochondrial subpopulations, indicating that extrapolation of findings should be made with caution and within the context of the underlying metabolic environment.

**Figure 1:**
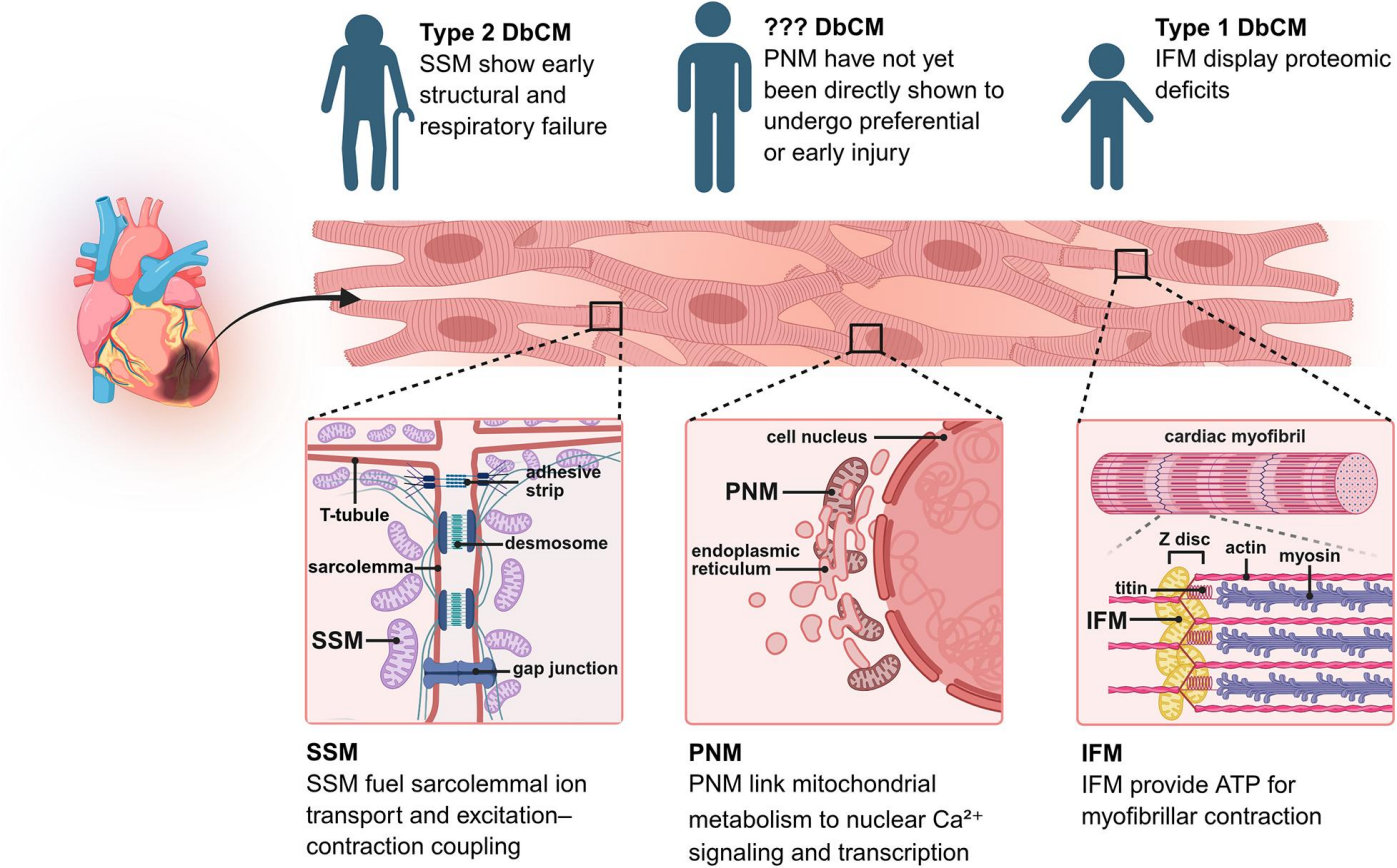
SSM near the cell membrane drive ion transport and contraction; PNM surround the nucleus, linking energy production to nuclear signaling; IFM between myofibers power contraction. SSM fail early in type 2 DbCM, PNM injury patterns remain unclear, and IFM show protein deficits in type 1 DbCM.

Beyond these classical SSM/IFM distinctions, PNM, mitochondria localized in close proximity to the nucleus, are increasingly recognized as a specialized subpopulation that functionally couples mitochondrial metabolism to nuclear Ca^2+^ signaling (35), redox-sensitive transcriptional regulation, and epigenetic remodeling (36). Their strategic positioning enables them to participate in mitochondria-to-nucleus retrograde signaling, including ROS-mediated activation of transcription factors (36) and modulation of nuclear Ca^2+^ transients (16). Emerging evidence suggests that PNM exhibit heightened sensitivity to Ca^2+^ overload and mitochondrial depolarization under stress conditions (37), potentially due to their proximity to nuclear Ca^2+^ release sites and distinct local microenvironment. This raises the possibility that PNM may act as early sensors or amplifiers of metabolic stress, initiating nucleus-directed transcriptional reprogramming that contributes to maladaptive remodeling in DbCM (Figure 1).

However, direct experimental evidence demonstrating preferential or temporally early PNM injury in diabetic hearts remains limited, highlighting an important gap in current knowledge. The lack of direct evidence for preferential or early PNM injury in diabetic hearts likely reflects technical and conceptual limitations rather than true biological absence. First, conventional mitochondrial isolation approaches, which effectively distinguish subsarcolemmal and interfibrillar populations, lack the spatial resolution to isolate PNM as a discrete fraction, thereby obscuring their specific molecular signatures. Second, although advanced imaging tools and genetically encoded sensors have improved the assessment of mitochondrial function, their application to simultaneously resolve subpopulation identity, membrane potential, and local Ca^2+^ dynamics in intact cardiomyocytes, particularly *in vivo*, remains challenging. Third, PNM are currently defined primarily by subcellular localization, as specific molecular markers uniquely identifying this population are lacking, limiting targeted manipulation. Finally, most studies in diabetic cardiomyopathy have focused on global mitochondrial dysfunction or SSM/IFM comparisons, with relatively little attention to mitochondria–nucleus signaling.

Resolving mitochondrial heterogeneity in diabetic cardiomyopathy requires an integrative, multi-scale toolkit. Subcellular fractionation combined with targeted respirometry has long demonstrated functional differences between subsarcolemmal and interfibrillar mitochondria in the heart, including in diabetic models, where selective impairment of interfibrillar mitochondrial oxidative capacity has been reported (29). Complementary mitochondrial proteomics and lipidomics studies have further revealed remodeling of electron transport chain components and cardiolipin composition under diabetic conditions, linking structural lipid alterations to impaired respiratory efficiency (33, 38, 39). In parallel, mtDNA sequencing approaches have identified increased mutation burden and altered copy number in metabolic disease, suggesting a genetic layer of mitochondrial heterogeneity (40). Ultrastructural analyses using high-resolution electron microscopy, including 3D reconstruction and FIB-SEM, have provided spatial evidence of mitochondrial fragmentation, cristae disruption, and region-specific damage in diabetic myocardium (41). More recently, emerging spatial transcriptomic approaches have begun to map localized stress and metabolic gene expression programs within cardiac tissue (42), offering a framework to connect subcellular mitochondrial defects with nuclear responses. These converging lines of evidence support a model in which mitochondrial dysfunction in diabetic cardiomyopathy is regionally and functionally heterogeneous rather than uniform. Vulnerable subpopulations—particularly those embedded within the contractile apparatus—may exhibit early deficits in bioenergetics, increased reactive oxygen species production, and impaired quality control, thereby acting as focal initiators of cellular dysfunction. This localized failure can propagate through disrupted mitochondrial dynamics and signaling, ultimately contributing to myocardial remodeling. Importantly, these insights also highlight potential therapeutic opportunities, such as stabilizing cardiolipin, modulating mitochondrial dynamics, or enhancing protein import and folding capacity in selectively vulnerable mitochondrial pools.

## Mitochondrial ROS dynamics and spatial signalling

Mitochondrial ROS generation is a central pathogenic event in DbCM. In cardiomyocytes, the mitochondrial electron transport chain (ETC) is the predominant source of mtROS, primarily at complex I and complex III, where excessive reducing pressure leads to electron leakage to oxygen, forming superoxide anions (O_2_·⁻). In diabetes, enhanced fatty acid oxidation and decreased glucose utilization elevate the NADH/FADH_2_ ratio and hyperpolarize the mitochondrial membrane, favoring both forward electron leak and reverse electron transport (RET) from the reduced ubiquinone pool back to complex I—a potent trigger of ROS bursts (43, 44). Metabolic remodeling further amplifies this process through accumulation of intermediates such as succinate, driving RET-dependent ROS generation.

Beyond the ETC, several mitochondrial-associated enzymes contribute to mtROS production. The redox adaptor p66Shc translocates to mitochondria under hyperglycemia and transfers electrons from cytochrome c to oxygen, producing hydrogen peroxide (45). Monoamine oxidases (MAO-A), located on the outer mitochondrial membrane, are upregulated in diabetic and obese hearts and generate substantial amounts of H_2_O_2_ during catecholamine metabolism (46). Meanwhile, mitochondrial antioxidant systems—such as manganese superoxide dismutase (MnSOD/SOD2), peroxiredoxins, and glutathione peroxidases—are often impaired. What's more, in diabetic cardiomyopathy, as in the failing heart, disruption of mitochondrial quality control—manifested both by imbalanced fusion–fission dynamics involving dynamin-related protein 1 (Drp1), mitofusins 1 (MFN1) and mitofusins 2 (MFN2) and by impaired PINK1–Parkin-mediated mitophagy—culminates in the accumulation of dysfunctional mitochondria and a feed-forward amplification of ROS (47–51). Notably, pharmacological modulation of mitophagy has emerged as a strategy to attenuate mtROS-driven cardiac injury. Levosimendan, for instance, limits excessive mitophagy via cyclic GMP-AMP synthase–stimulator of interferon genes (cGAS–STING) inhibition (52), whereas diltiazem hydrochloride promotes BNIP3L/NIX-mediated clearance of damaged mitochondria (53).

**Figure 2:**
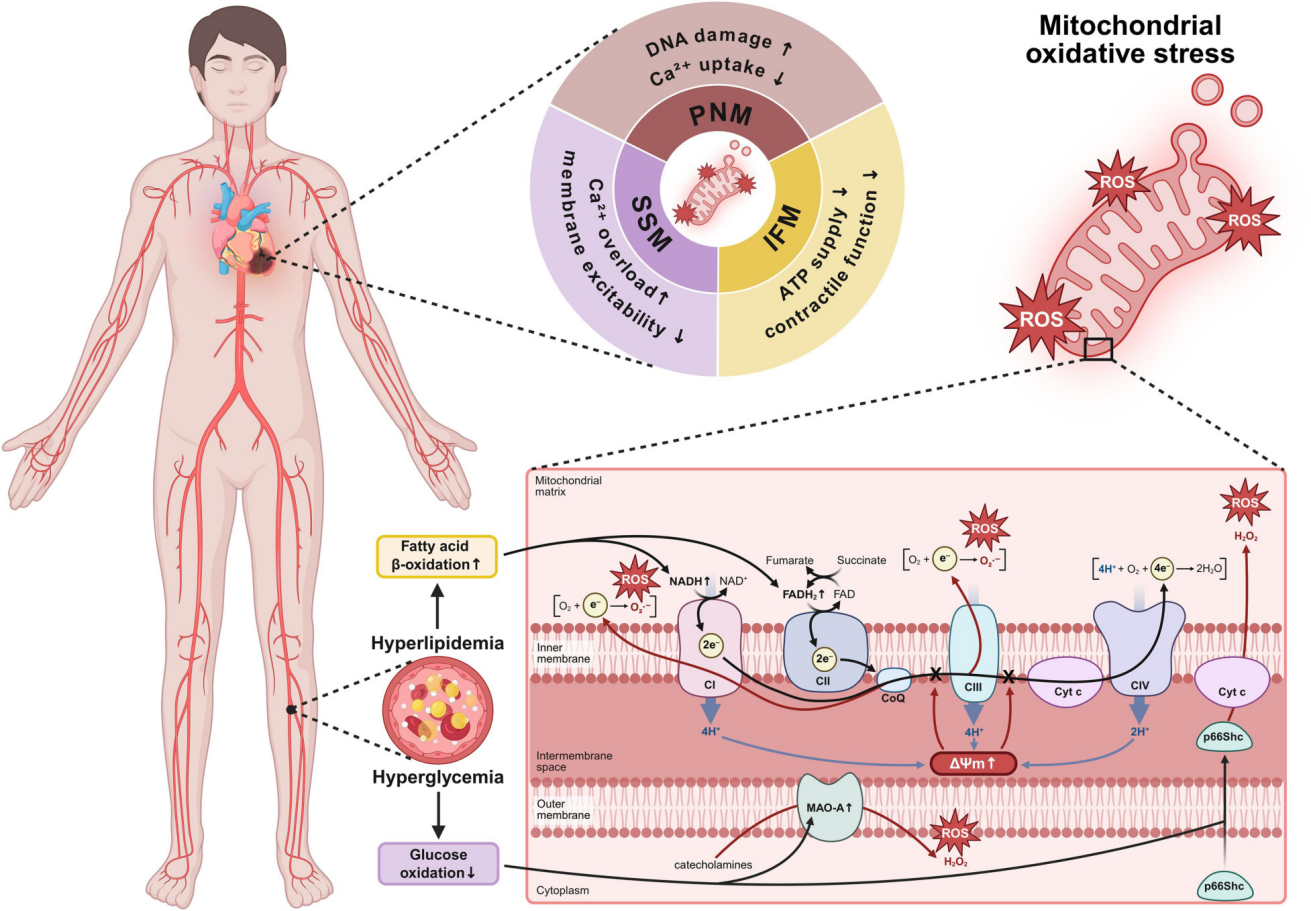
This schematic depicts the spatial distribution of SSM, IFM, and PNM mitochondria in cardiomyocytes, underscoring their distinct roles in ROS generation and functional impairments. Metabolic remodeling characterized by enhanced fatty acid oxidation and reduced glucose utilization elevates the NADH/FADH_2_ ratio, promoting ETC dysfunction at complexes I/III and RET via a hyperpolarized mitochondrial membrane. This process, amplified by increased ROS production from enzymes such as MAO-A and p66Shc, induces oxidative damage including DNA lesions, Ca^2+^ overload, and ATP depletion. SSM, IFM, and PNM exhibit differential susceptibilities to Ca^2+^ overload and mPTP opening, with PNM uniquely regulating nuclear redox signaling. Collectively, these abnormalities disrupt cardiac homeostasis through a feed-forward cycle of mitochondrial dysfunction and oxidative stress, highlighting the convergence of metabolic and redox perturbations in diabetic cardiomyopathy pathogenesis.

SSM, IFM, and PNM differ in their coupling to local Ca^2+^ handling, ATP demand and redox buffering, thereby shaping both their propensity to generate ROS and the local signaling reach of those ROS. SSM display distinct sensitivity to Ca^2+^ overload and mitochondrial permeability transition pore (mPTP) opening; therefore, their ROS are well placed to perturb sarcolemmal ion handling and membrane excitability under metabolic stress (54). IFM, characterized by relatively high Ca^2+^ uptake capacity and oxidative throughput, are tightly coupled to sarcomeric ATP supply; oxidative impairment of IFM preferentially undermines contractile energetics and excitation–contraction coupling (16). PNM are well positioned to deliver redox and Ca^2+^ signals to the nucleus; perinuclear clustering can create an oxidant-rich nuclear microdomain and PNM from failing cardiomyocytes show increased susceptibility to mtROS generation and impaired Ca^2+^ uptake—features consistent with an enhanced ability to influence transcriptional and DNA-damage/epigenetic responses (8) (Figure 2). Direct experimental comparisons between cardiac mitochondrial subpopulations have demonstrated quantifiable differences in respiratory capacity, Ca^2+^ handling, and stress responsiveness (8). For example, IFM exhibit significantly higher state 3 respiration than SSM under physiological conditions, whereas pressure overload preferentially depresses IFM respiratory capacity, eliminating this difference in heart failure models (55). In parallel, SSM display greater susceptibility to Ca^2+^-induced inhibition of oxidative phosphorylation and lower Ca^2+^ retention capacity, indicating enhanced sensitivity to permeability transition compared with IFM (54). Additional studies further demonstrate subpopulation-specific differences in electron transport chain activity and substrate utilization during ischemia–reperfusion, highlighting distinct metabolic adaptations between SSM and IFM (56).

Therapeutically, these findings argue against indiscriminate global ROS scavenging in DbCM and favour precision strategies aimed at stabilizing vulnerable mitochondrial pools or restoring local redox signaling. Clinical translation will require demonstration of such subpopulation-specific dysfunction in human myocardium and validation of safety/effectiveness in translational models.

## Innate immune activation by mitochondrial damage

Mitochondrial injury forms a mechanistic bridge between metabolic stress and sterile innate immunity in DbCM (57); however, this process is unlikely to be spatially uniform across the cardiomyocyte. Instead, emerging evidence suggests that mitochondrial subpopulations may differentially contribute to immunogenic signalling depending on their subcellular localization and functional properties.

SSM, which are positioned beneath the plasma membrane, are exposed to higher fluctuations in extracellular nutrient supply, lipid intermediates and cytosolic Ca^2+^ (15). Under hyperglycaemic and lipotoxic conditions, SSM exhibit enhanced oxidative stress and membrane instability, which may favour early mtDNA oxidation and release into the cytosol (58). In contrast, IFM, embedded within the contractile apparatus, are more tightly coupled to ATP production and excitation–contraction coupling (16, 59); their damage may preferentially impair bioenergetics rather than directly initiate immunogenic signalling. PNM, located in close proximity to the nucleus, are uniquely positioned to influence nuclear signalling (37) and may facilitate efficient coupling between mtDNA release and activation of nuclear-directed innate immune pathways, although direct experimental evidence for this remains limited.

mtDNA can exit mitochondria through multiple mechanisms, including transient mitochondrial permeability transition pore (mPTP) opening, BAX/BAK-mediated outer membrane permeabilization, and defective mitophagy that permits persistence of damaged mitochondrial fragments (57, 60). Importantly, these release routes may not be equivalent across mitochondrial subpopulations. For example, mitochondria experiencing high Ca^2+^ fluxes and oxidative stress, conditions that are more typical of SSM, may preferentially undergo mPTP-dependent release (54), whereas impaired mitophagic clearance of structurally constrained IFM could lead to delayed but sustained leakage of mitochondrial components. These distinctions are likely to influence the quantity, oxidation state and intracellular distribution of mtDNA, thereby shaping its immunogenic potential.

Once in the cytosol, double-stranded mtDNA acts as a damage-associated molecular pattern that activates the cGAS–STING pathway (61). Given the spatial organization of cardiomyocytes, mtDNA derived from PNM may have a higher probability of interacting with perinuclear pools of cGAS and downstream signalling machinery, potentially amplifying interferon responses and transcriptional reprogramming. In contrast, mtDNA released from peripheral SSM may preferentially engage cytosolic or membrane-associated signalling complexes, contributing to localized inflammatory responses in the sarcolemmal or vascular interface. Although this spatial model is conceptually supported by the intracellular organization of both mitochondria and innate immune sensors, direct evidence demonstrating subpopulation-specific coupling to cGAS–STING activation in the heart is currently lacking.

In parallel, mtROS and oxidized mtDNA contribute to activation of the NLRP3 inflammasome (62–64). SSM, which generally exhibit lower antioxidant capacity and higher susceptibility to oxidative stress, may represent a dominant source of mtROS-driven inflammasome priming. Oxidized mtDNA released from these mitochondria can bind to NLRP3 and promote inflammasome assembly, leading to caspase-1 activation and maturation of IL-1β and IL-18 (65, 66). By contrast, IFM-derived signals may play a more secondary or sustained role, particularly under conditions of chronic mitochondrial dysfunction. Whether distinct mitochondrial subpopulations differentially regulate inflammasome activation kinetics or magnitude in cardiomyocytes remains an open question.

Mitochondria-derived vesicles (MDVs) and extracellular vesicles containing mitochondrial components (mito-EVs) provide an additional layer of spatial regulation. Under mild stress, MDVs may facilitate selective removal of damaged cargo, thereby limiting immune activation (67). However, under conditions of overwhelming mitochondrial injury, vesicles derived from different mitochondrial regions may carry oxidized mtDNA and proteins to endolysosomal compartments or extracellular space, enabling intercellular propagation of inflammatory signals (68, 69). Whether MDV biogenesis differs between SSM, IFM and PNM, and how this influences immune signalling, has not yet been systematically investigated.

Preclinical studies in diabetic and lipotoxic models demonstrate activation of cGAS–STING and NLRP3 pathways in the heart, together with increased cytosolic mtDNA and inflammatory cytokine production (61). However, these studies generally do not distinguish between mitochondrial subpopulations, limiting mechanistic resolution. In human DbCM, transcriptomic analyses reveal enrichment of interferon-responsive and inflammasome-related pathways (70), but direct evidence linking specific mitochondrial subpopulations to innate immune activation in human myocardium remains scarce.

**Figure 3:**
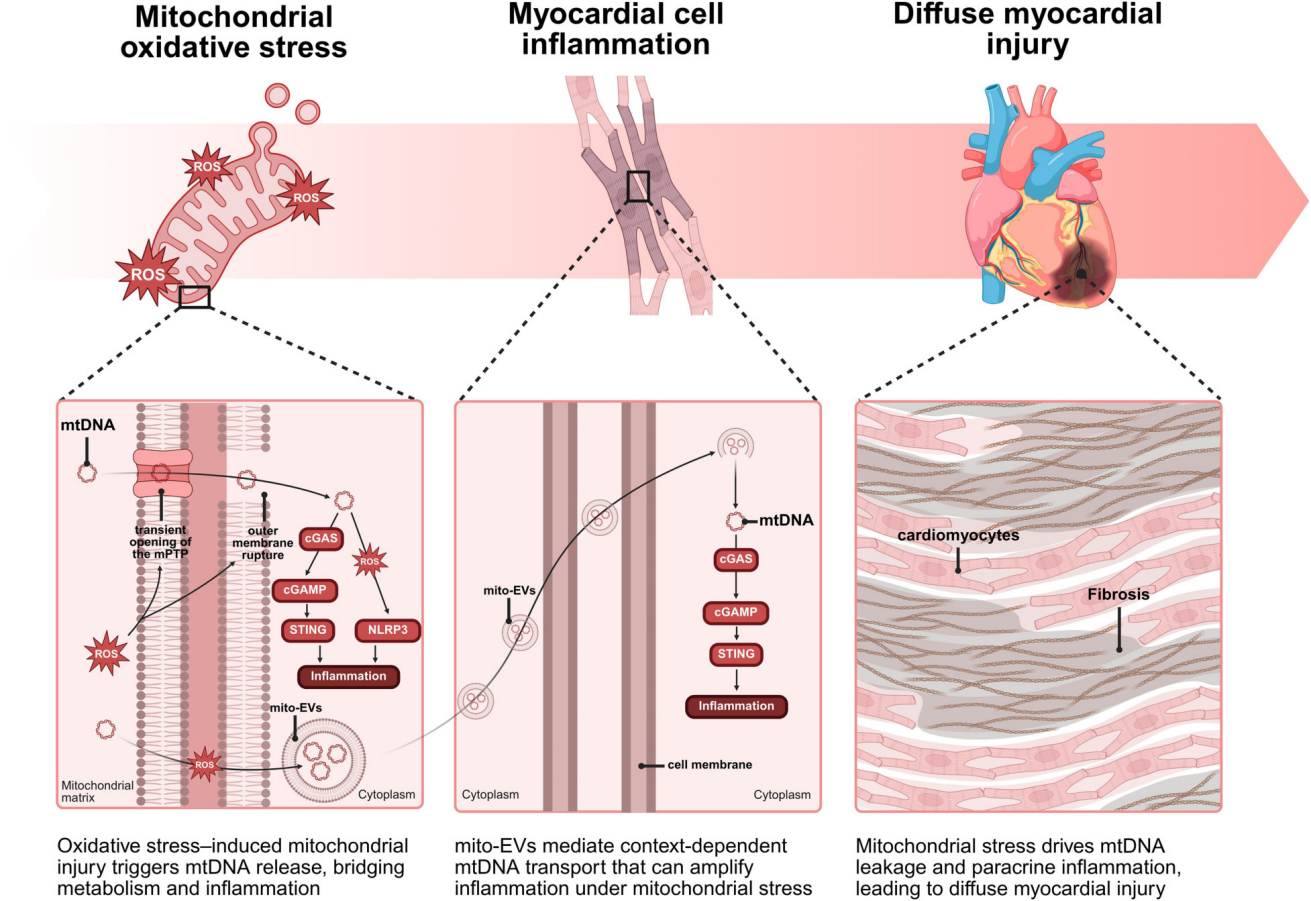
Mitochondrial oxidative stress and dysfunction trigger multi-route release of mtDNA. Released mtDNA acts as a DAMP, activating cGAS-STING and NLRP3 inflammasome pathways, ultimately driving cardiomyocyte fibrosis and maladaptive cardiac remodeling in DbCM.

Collectively, these observations support a model in which mitochondrial damage drives innate immune activation in DbCM (Figure 3), but also highlight a critical knowledge gap: the spatial origin of immunogenic mitochondrial signals within the heterogeneous mitochondrial network is largely undefined. Addressing this question will be essential to refine our understanding of immunometabolic coupling and to enable more precise therapeutic targeting.

## Translational roadmap

Translating mitochondria-targeted therapies from concept to clinic requires a staged, mechanism-driven pipeline that establishes spatially resolved preclinical signatures of treatment responsiveness, validates biomarkers and target engagement in small human proof-of-concept (PoC) studies, tests adaptive, biomarker-enriched Phase II designs to refine dosing and selection, and advances to definitive randomized controlled trials with pre-specified enrichment criteria and long-term safety monitoring.

Preclinical validation must capture subcellular heterogeneity of mitochondrial injury and the mechanisms targeted by the intervention. Recommended assays include subcellular fractionation to separate interfibrillar and subsarcolemmal mitochondria, high-resolution respirometry on fresh or mechanically permeabilized cardiac tissue to quantify OXPHOS capacity and respiratory control, simultaneous measurement of mitochondrial H_2_O_2_ production, and targeted lipidomics to profile cardiolipin species that determine cristae stability. These methods have been adapted successfully to small human cardiac samples and animal models and provide reproducible, mechanistic endpoints for predicting response (54, 71, 72).

Early human PoC studies should be small, randomized, and biomarker-guided to demonstrate target engagement and safety rather than hard clinical endpoints. Candidate biomarkers and surrogate endpoints include: myocardial mitochondrial oxygen consumption (by high-resolution respirometry on percutaneous endomyocardial biopsy or optimized tissue homogenate protocols); *in vivo* myocardial energetics (³¹P-MRS for PCr/ATP ratios) and fibrosis/metabolic imaging by multiparametric cardiac MRI; and circulating mitochondrial biomarkers such as total and oxidized cell-free mtDNA or mtDNA copy-number signatures. Multi-omics approaches are now widely applied to investigate metabolic and cardiovascular conditions (73, 74), pointing to the potential utility of similar integrative strategies for biomarker discovery in diabetic cardiomyopathy. Emerging studies show circulating mtDNA signatures vary in cardiometabolic patients and correlate with disease states, supporting their use for stratification. PoC trials should use these readouts to define biological responders (75–77).

An adaptive design allows refinement of dose/timing and enrichment rules based on interim biomarker response (e.g., a pre-specified reduction in tissue mtROS signal or improvement in myocardial energetic ratio). Statistical frameworks for biomarker-guided adaptive enrichment are available and can control type I error while increasing the chance of identifying a responsive subgroup. Practical precedents include adaptive trials in mitochondrial disease and other metabolic interventions. Endpoints should combine mechanistic biomarkers (mitochondrial OCR, oxidized mtDNA, cardiolipin profile) with physiological measures (diastolic function, exercise capacity) and safety monitoring (78, 79).

Definitive trials should prospectively stratify or enrich participants using the validated biomarker signature (for example, patients with elevated circulating oxidized mtDNA and diminished myocardial energetics). Primary outcomes may include heart-failure hospitalization, change in diastolic function by echocardiography/CMR, and patient-centered outcomes. Because mitochondrial interventions may affect host defense, adaptive stress responses, or long-term metabolism, extended safety follow-up (including infection rates, cancer signals, and metabolic endpoints) is required (80, 81).

## Conclusion

By emphasizing mitochondrial subpopulation-specific vulnerabilities and the spatiotemporal dynamics of mtROS, this review moves beyond the traditional paradigm of global mitochondrial dysfunction. This integrative framework not only refines our mechanistic understanding of DbCM but also outlines a rationale for precision-targeted therapeutic approaches, setting our perspective apart from prior reviews.
